# Effect of essential oils against acaricide‐susceptible and acaricide-resistant *Rhipicephalus* ticks

**DOI:** 10.1007/s10493-021-00601-x

**Published:** 2021-02-24

**Authors:** Darcy Adriann Rebonato Luns, Renato Martins, Sofia Pombal, Jesus M. Lopez Rodilla, Naftaly W. Githaka, Itabajara da Silva Vaz, Carlos Logullo

**Affiliations:** 1 Laboratório Integrado de Bioquímica Hatisaburo Masuda and Laboratório de Bioquímica de Artrópodes Hematófagos, NUPEM - UFRJ, Campus Macaé, Avenida São José do Barreto, São José do Barreto, Macaé, RJ CEP 27965-045 Brazil; 2 Departamento de Química, Materiais Fibrosos e Tecnologias Ambientais - FibEnTech. Universidade da Beira Interior, Rua Marques de Ávila e Bolama, 6201-001 Covilhã, Portugal; 3 Tick Unit, International Livestock Research Institute, P.O. Box 30709, Nairobi, Kenya; 4 Centro de Biotecnologia - UFRGS, Av. Bento Gonçalves 9500, Prédio 43421, Campos do Vale, C.P. 15005, Porto Alegre, RS CEP 91501-970 Brazil

**Keywords:** *Rhipicephalus* spp, Tick, Essential oil, *Schinus mole*, *Bulnesia sarmientoi*, Acaricide

## Abstract

The indiscriminate use of acaricides is a problem worldwide and has increased the selection of acaricide-resistant tick populations. The goal of this study was to evaluate the acaricide effects of two essential oils (from *Schinus molle* and *Bulnesia sarmientoi*) using the larval immersion test on three *Rhipicephalus* tick species. *Rhipicephalus evertsi*, *Rhipicephalus appendiculatus* and *Rhipicephalus pulchelus* ticks collected in Kenya, without history of acaricide exposure, were tested, as well as individuals from two populations of *Rhipicephalus microplus* (with or without history of acaricide exposure), for comparison. The sample most resistant to the treatments was a population of *R. microplus* with previous acaricide exposure, whereas the least tolerant sample was a strain of the same species that never had contact with acaricides (Porto Alegre strain). Interestingly, the field tick samples without previous acaricide exposure responded to essential oils with a mortality profile resembling that observed in the acaricide-resistant *R. microplus* field population, and not the susceptible Porto Alegre strain. The essential oil of *B. sarmientoi* and its two components tested (guaiol and bulnesol) caused the highest mortality rates in the tested species and are potential molecules for future studies on control methods against these species.

## Introduction

Ticks are important disease vectors, affecting both human and animal populations, and transmitting a wide range of pathogens (Silva and Silva 1999; Corson et al. 2004; Sonenshine and Roe 2006). These arthropods cause considerable losses to the cattle industry worldwide (Grisi et al. 2014). Infestation generates several deleterious effects on the host including blood loss, reduced weight gain and milk production, and skin damage at the site of attachment (Fular et al. 2018; Souza Conceição et al. 2017; Souza et al. 2012).

In addition to the direct effects caused by blood feeding, ticks also transmit pathogenic organisms of great economic importance. The bovine tick *Rhipicephalus microplus* is the main vector of Babesia protozoans (*Babesia bovis* and *Babesia bigemina*) and the bacterium *Anaplasma marginale* (Barbosa et al. 2013; Souza Conceição et al. 2017). *Rhipicephalus appendiculatus* is the main vector of the protozoan *Theileria parva*, the agent of East Coast fever in cattle, a clinical syndrome of great economic importance in 12 sub-Saharan countries (Nene et al. 2016). *Rhipicephalus evertsi* is the vector of *Rickettsia africae* (Hedimbi et al. 2011; Walker et al. 2014), whereas *Rhipicephalus pulchellus* transmits benign bovine theileriosis caused by *Theileria taurotragi*, in addition to sheep's milk virus. To humans, these ticks can transmit *Rickettsia conorii*, a bacterium that causes tick typhus, as well as the Crimean-Congo hemorrhagic fever virus (Walker et al. 2014).

The most common method for tick control is the use of synthetic acaricides, however, the misuse and overuse use of these products has accelerated the selection of resistant tick populations (Daher 2011; Souza et al. 2012; Biegelmeyer et al. 2014). An important strategy explores the use of bioactive compounds from plants to develop new acaricides (Roh et al. 2003; Santos et al. 2012a, b; Štefanidesová et al. 2017; Rodriguez-Vivas et al. 2018; Vinturelle et al. 2017). Research on plant bioactive compounds can lead to the identification of new molecules presenting different modes of action, biological targets and synergy in comparison to current commercial products (Benelli and Pavela 2018; Pavela 2015; Rattan 2010; Agwunobi et al. 2020).

*Bulnesia sarmientoi* and *Schinus molle* are plants native to South America belonging to the order Sapindales. The essential oils of the two species present high antimicrobial activity (Abd-allah et al. 2015; Pegard 2015), fungicidal activity (Castillo et al. 2012; Pawlowski et al. 2012) and repellent and insecticidal actions against several pests (De Batista et al. 2016; Ferrero et al. 2006; Rizwan-ul-Haq and Aljabr 2015; Rodilla et al. 2011; Torres et al. 2012). These properties, associated with low toxicity to mammals (Martins et al. 2014), may contribute to the development of new products to control infestations by different arthropods of economic importance.

The present work investigated the effect of essential oils from *B. sarmientoi* and *S. molle* on larvae from four *Rhipicephalus* species of economic importance. These oils and the two main components of both (α-phellandrene and sabinene of *S. mole*, bulnesol and guaiol of *B. sarmientoi*) may have potential for future studies on control methods against these species.

## Materials and methods

### Essential oils extraction and analysis

#### Schinus molle

*Schinus molle* is a tree native to the Peruvian Andes and its resin is exuded from the trunk to obtain a liquid rubber that was used for embalming (Adorno and Boserup 2016). The leaves of this species are composed and have serrated margins, the yellow flowers are gathered in inflorescences and its fruits show reddish color of the drupe type (IBF 2021). Fruits and leaves were processed by steam distillation in the Clevenger apparatus according to the European Pharmacopeia. The resulting sample was analyzed and components were identified by GC and GC–MS. Polar products not detectable by GC–MS were isolated by CC and identified by ^1^H-NMR, ^13^C-NMR and high-resolution mass spectrometer.

The chemical composition of the essential oil was previously reported and consists mainly of monoterpene hydrocarbons (e.g., α-pinene 5.32%, β-pinene 4.50%, sabinene 34.77%, limonene 4.18, terpinen-4-ol 5.50%, β-caryophyllene 3.84%, and other), and some sesquiterpenes such as (+) spathulenol 3.91% and germacrene-D 7.06%. The chromatographic profile of the sample was consistent with the ranges defined by standard IRAM (Argentina) for the essential oil of the fruits and leaves of *S. molle.*

Two of the essential oil components, the monoterpenoids α-phellandrene and sabinene, were selected based on previous functions described (Espinosa-García and Lanhenheim 1991; Zhang et al. 2017) and purchased from Sigma-Aldrich and kept as a 20% (w/v) stock solution for further tests. The essential oil and its components were solubilized in methanol 100%.

#### Bulnesia sarmientoi

*Bulnesia sarmientoi* commonly known as sacred wood (palo santo in Spanish) is a tree that grows in woods in the region of Chaco, belonging to a portion of the territory of Argentina and Paraguay. The leaves of this plant are short, light green and oval. Its small flowers are white and the fruits yellow-orange. The trunk is protected by a thick gray bark, traditionally used as incense due to its aromatic property. *B. sarminetoi* essential oil is extracted from wood and sawdust by steam distillation. The obtained oil has a yellowish-green coloration, containing a high concentration of two sesquiterpene alcohols: bulnesol and guaiol (Rodilla et al. 2011).

Essential oil from *B. sarmiento*i was extracted using vapor distillation of wood by the Cooperative Colonizing Society Chortitzer Komitee (Loma Plata, Paraguay). The essence of palo santo was dissolved in n-hexane with mild heating. This solution was allowed to cool and placed in a refrigerator to form guaiol crystals, which were then harvested by filtering. From the remaining solution, mother liquor, the solvent was evaporated to obtain the essential oil with low guaiol content. The product was chromatographed on flash chromatography with hexane and hexane–ethyl acetate mixtures of increasing polarity. This system allowed us to obtain different fractions with mixtures of minor products, and fractions containing pure guaiol and bulnesol that were used for the tests in this work (Rodilla et al. 2011).

### Ticks

*Rhipicephalus microplus* from acaricide susceptible strain named Porto Alegre was reared in bovines, which were brought from a tick-free area and maintained in insulated individual boxes at Faculdade de Veterinária of Universidade Federal do Rio Grande do Sul, Porto Alegre, Brazil. Calves between 5 and 10 months old were infested with 15-day-old tick larvae. Twenty-two days after infestation, female ticks were collected from calves (Reck et al. 2009). Animal care was conducted in accordance with institutional guidelines (institutional approval number 27559).

For *R. microplus* field populations samples, fully engorged females were collected from cattle in a farm in Campos dos Goytacazes, Rio de Janeiro, Brazil (21° 45′ 21″ S, 41° 19′ 57″ W). In this farm, the acaricides cypermethrin, chlorpyrifos, fenthion and fipronil have been extensively used during the last 3 years for tick control.

The African ticks *R. evertsi*, *R. appendiculatus* and *R. pulchellus* were obtained from the tick colony of the Tick Vector Laboratory of International Livestock Research Institute (ILRI) in Nairobi, Kenya. These ticks were originally collected from field animals without history of acaricide treatment, and posteriorly maintained under standard laboratory conditions in the absence of acaricide exposure for multiple generations.

### Larval immersion test

Collected females were maintained in climatic chambers at 28 ºC and 80% relative humidity for oviposition. Eggs were collected every day in microtubes closed with a thin screen (10 mg per tube; i.e., approx. 200 eggs/tube). Hatching occurred approximately 21 days after the onset of oviposition. Bioassays were performed in 7- to 10-day-old larvae.

The larval immersion test (LIT) was chosen due to its higher sensitivity in comparison to other methods (Castro-Janer et al. 2010, 2011). Compounds were tested at the following concentrations: 0.01, 0.1, 1 and 10% (v/v). Ten days after egg hatching, about 1.5 mL of the test solutions were added to the tube until larvae were totally immersed. After 5 min, the liquid was removed, larvae were transferred to filter paper to remove excess solution, then placed in Petri dishes for 24 h. Mortality was evaluated by counting live larvae after this period. Methanol was used as solvent along with essential oils in the LIT.

### Statistical analysis

The results represent means based on experiments performed in triplicate (independent samples), according to Shaw (1966). Effect of treatments on larval mortality was analyzed by one-way ANOVAs using the SAS statistical software (SAS Institute, Cary, NC, USA). Tukey’s test was used for post-hoc analysis (α = 0.05).

## Results

### Mortality of *Rhipicephalus microplus* larvae from acaricide-exposed or non-exposed populations

**Fig. 1:**
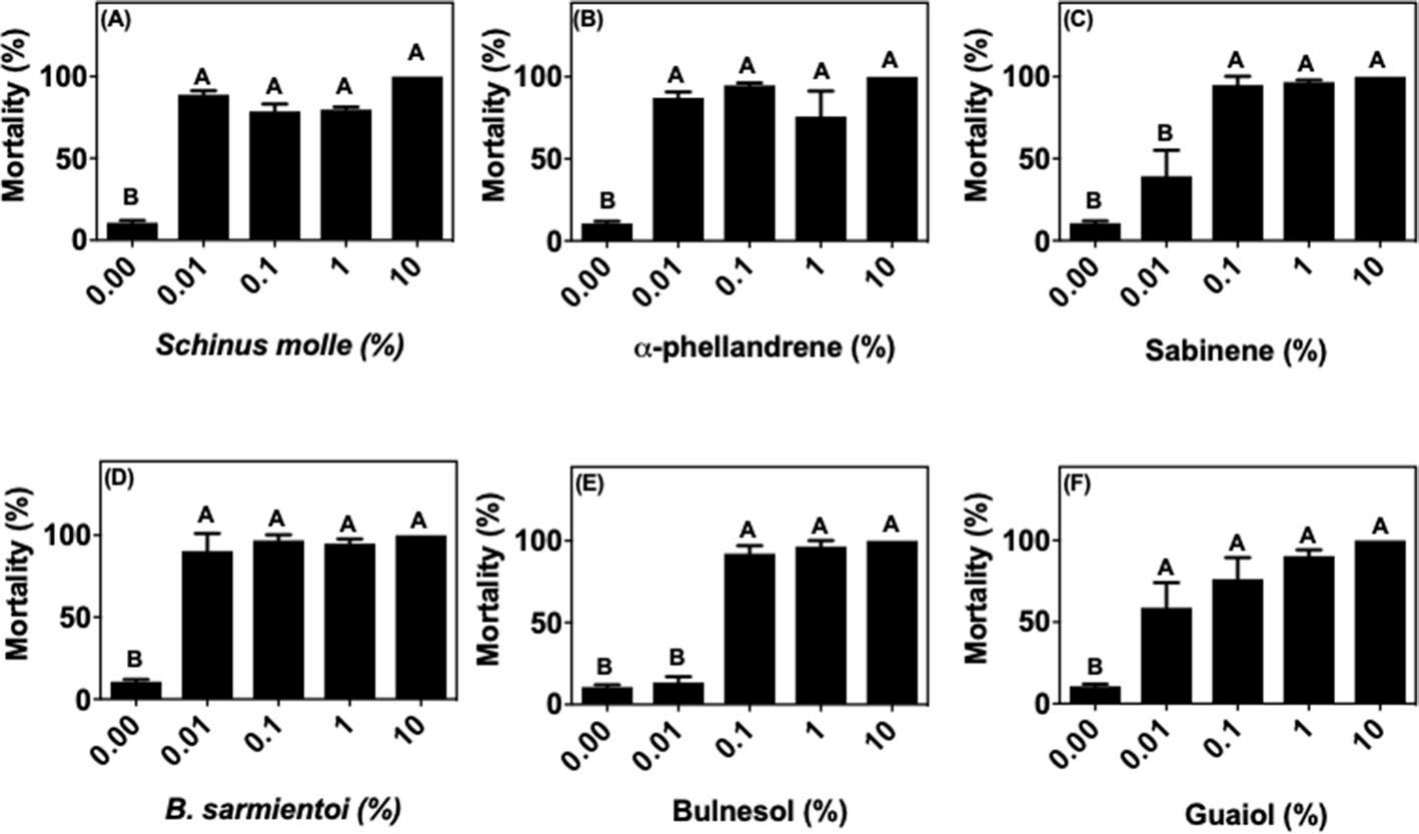
Mean (+ SD; n = 3) mortality of larvae from acaricide-susceptible *Rhipicephalus microplus* (Porto Alegre strain) after exposure to essential oils and their main components. **a** *Schinus molle* essential oil, **b** α-phellandrene, **c** sabinene, **d** *Bulnesia sarmientoi* essential oil, **e** bulnesol, and **f** guaiol. Means capped with the same letter are not significantly different (Tukey test: p > 0.05)

**Fig. 2:**
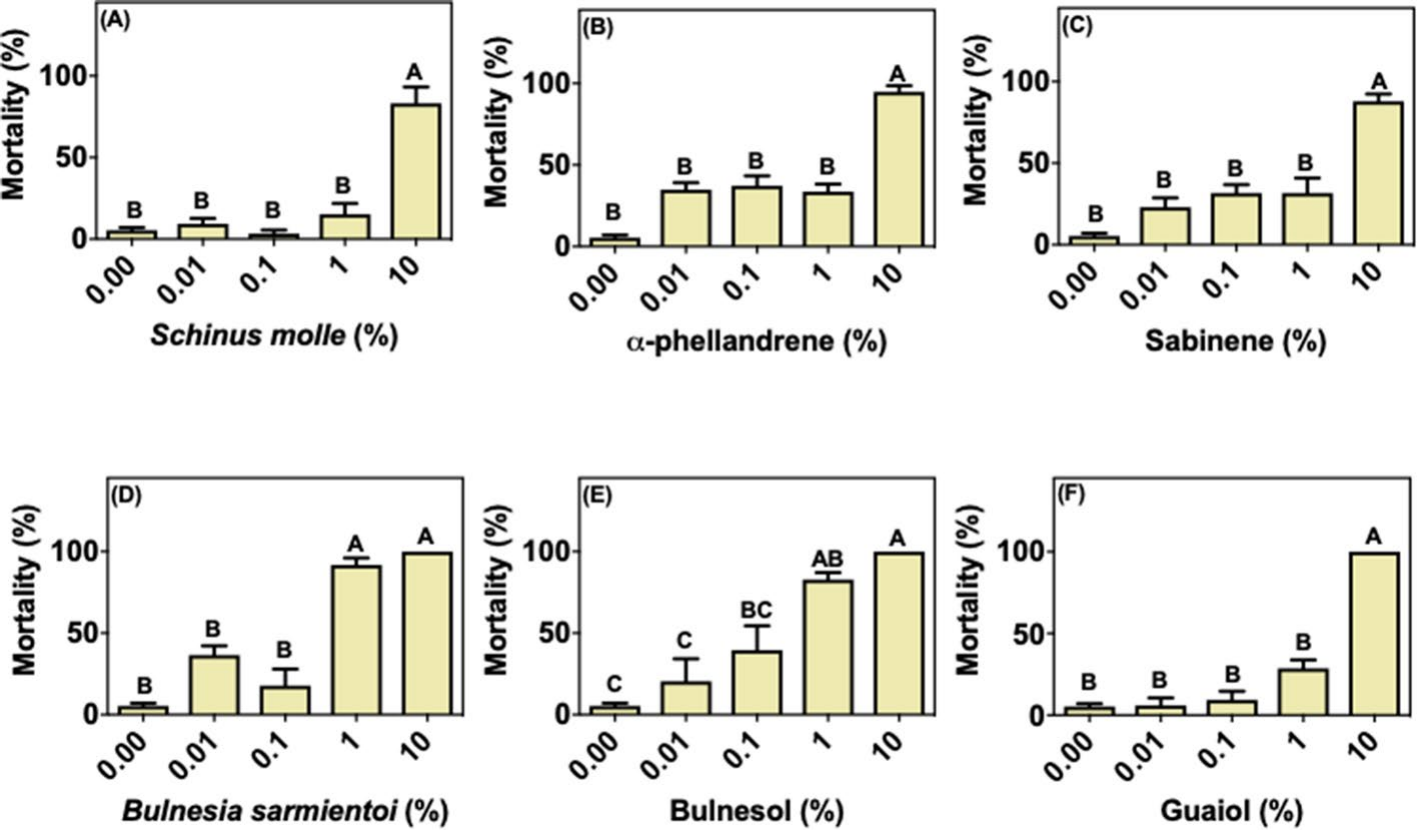
Mean (+ SD; n = 3) mortality of larvae from acaricide-resistant *Rhipicephalus microplus* field population after exposure to essential oils and their main components. **a** *Schinus molle* essential oil, **b** α-phellandrene, **c** sabinene, **d** *Bulnesia sarmientoi* essential oil, **e** bulnesol, and **f** guaiol. Means capped with the same letter are not significantly different (Tukey test: p > 0.05)

Both oils and the isolated compounds caused almost 100% mortality in larvae of *R. microplus* from the susceptible Porto Alegre strain at concentrations above 0.1% (Fig. 1). At 0.01%, sabinene, bulnesol and guaiol induced 40, 15 and 50% larval mortality, respectively, whereas all other compounds reached 100% at this concentration. The *R. microplus* field population has been exposed at least for 3 years to acaricides like cypermethrin, chlorpyrifos, fenthion and fipronil. Larvae from this population could better tolerate all treatments compared with the Porto Alegre strain (Fig. 2). Immersion of larvae in *S. molle* essential oil, α-phellandrene, sabinene, and guaiol at concentrations below 10% did not show statistically significant changes in mortality compared to control (Fig. 2a–c, e). *Bulnesia sarmientoi* essential oil and bulnesol induced mortality at 1% concentration (Fig. 2d, e). The mortality induced by guaiol was markedly different between acaricide-susceptible ticks and the field population, being effective at 0.01% against larvae from Porto Alegre strain but only at 10% concentration against field ticks (Figs. 1f and 2f).

### Mortality of *Rhipicephalus* spp. larvae from Kenyan field populations

**Fig. 3:**
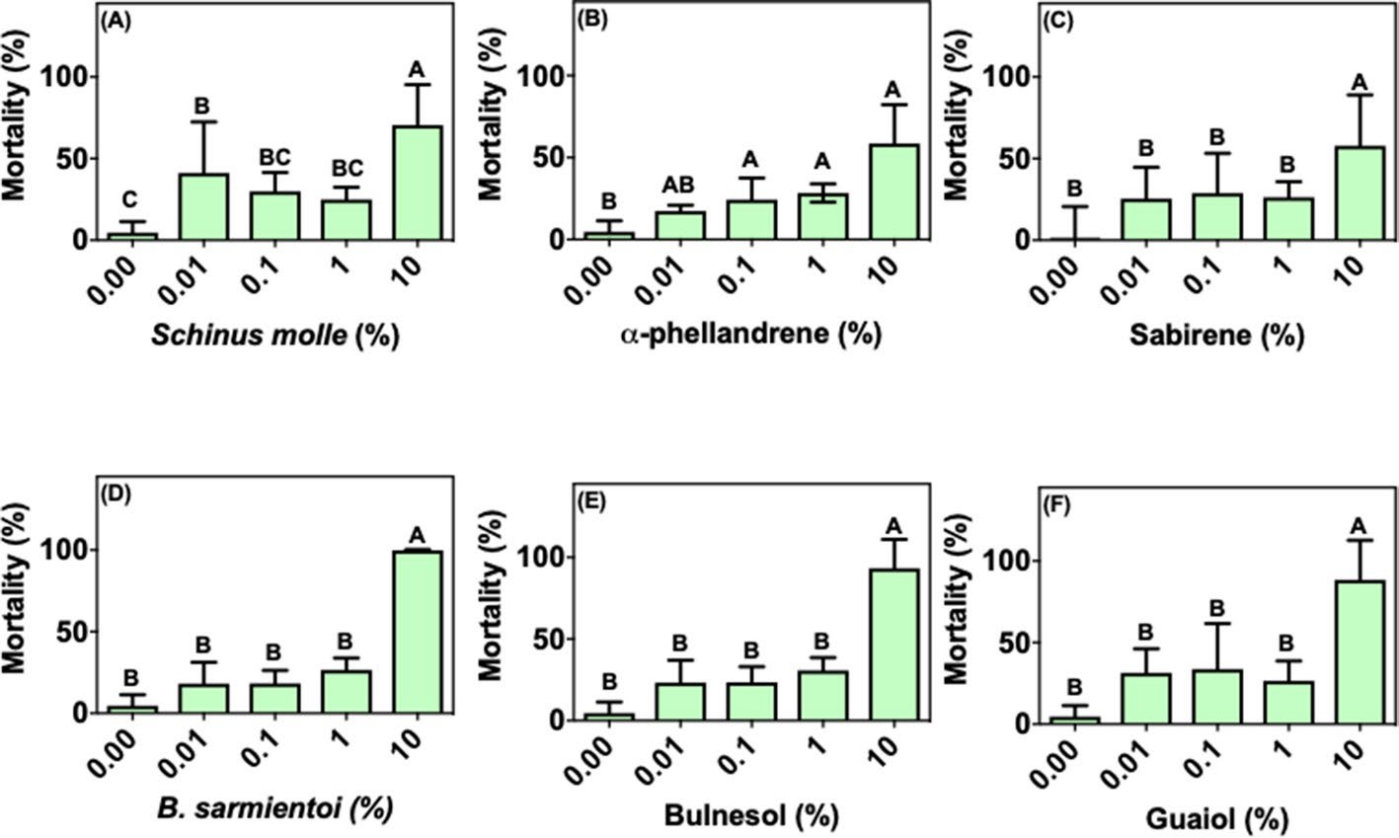
Mean (+ SD; n = 3) mortality of larvae from *Rhipicephalus evertsi* African field population after exposure to essential oils and their main components. **a** *Schinus molle* essential oil, **b** α-phellandrene, **c** sabinene, **d** *Bulnesia sarmientoi* essential oil, **e** bulnesol, and **f** guaiol. Means capped with the same letter are not significantly different (Tukey test: p > 0.05)

**Fig. 4:**
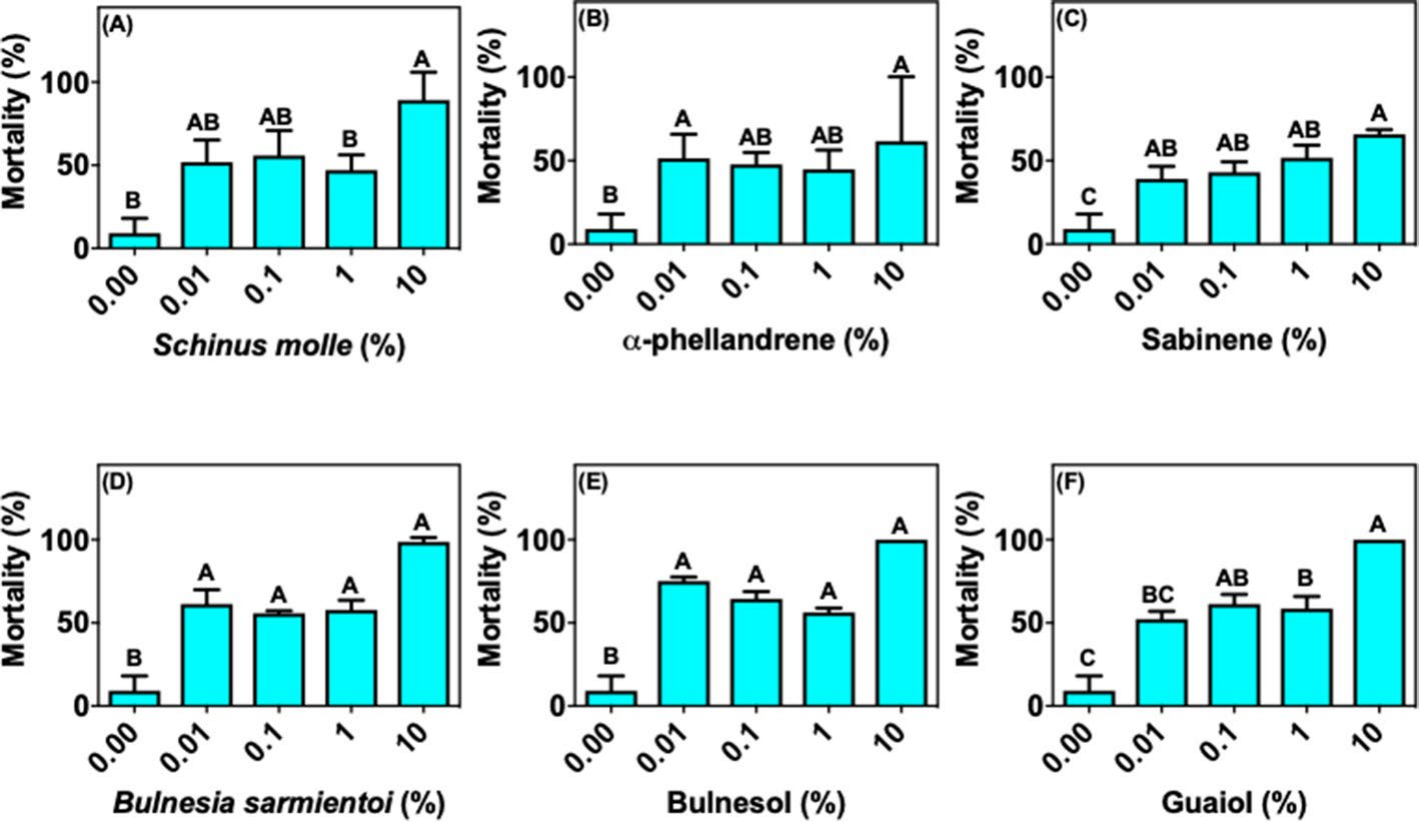
Mean (+ SD; n = 3) mortality of larvae from *Rhipicephalus appendiculatus* African field population after exposure to essential oils and their main components. **a** *Schinus molle* essential oil, **b** α-phellandrene, **c** sabinene, **d** *Bulnesia sarmientoi* essential oil, **e** bulnesol, and **f** guaiol. Means capped with the same letter are not significantly different (Tukey test: p > 0.05)

For *R. evertsi*, sabinene, *B. sarmientoi* essential oil, bulnesol or guaiol at concentrations up to 1% did not affect the larval mortality rate compared to control (Figs. 3c–f). In the case of *S. molle* essential oil and α-phellandrene, the mortality rates were < 50% in treatment with doses < 1% (Fig. 3a, b). *Bulnesia sarminetoi* essential oil, as well as its major components bulnesol and guaiol, showed 100% mortality only at the highest concentration tested (10%) (Fig. 3d–f). In contrast, *S. molle* essential oil at 10% presented a weaker larvicidal effect, between 58 and 70% (Fig. 3a). For *R. appendiculatus*, *S. molle* essential oil, α-phellandrene and sabinene treatments at the three lowest concentrations tested did not exceed 60% mortality rate (Fig. 4a–c). *Schinus molle* essential oil components, α-phellandrene and sabinene, were less effective compared with the oil; at the highest concentration tested (10%), they induced approximately 90% larval mortality. *Bulnesia sarminetoi* essential oil reached 60% mortality at the lowest concentration (Fig. 4d), whereas isolated bulnesol caused 80% mortality at the same dose (Fig. 4e). *Bulnesia sarmientoi* essential oil at 10%, as well as bulnesol and guaiol at 10%, caused 100% mortality of the immersed larvae (Fig. 4d–f).

**Fig. 5:**
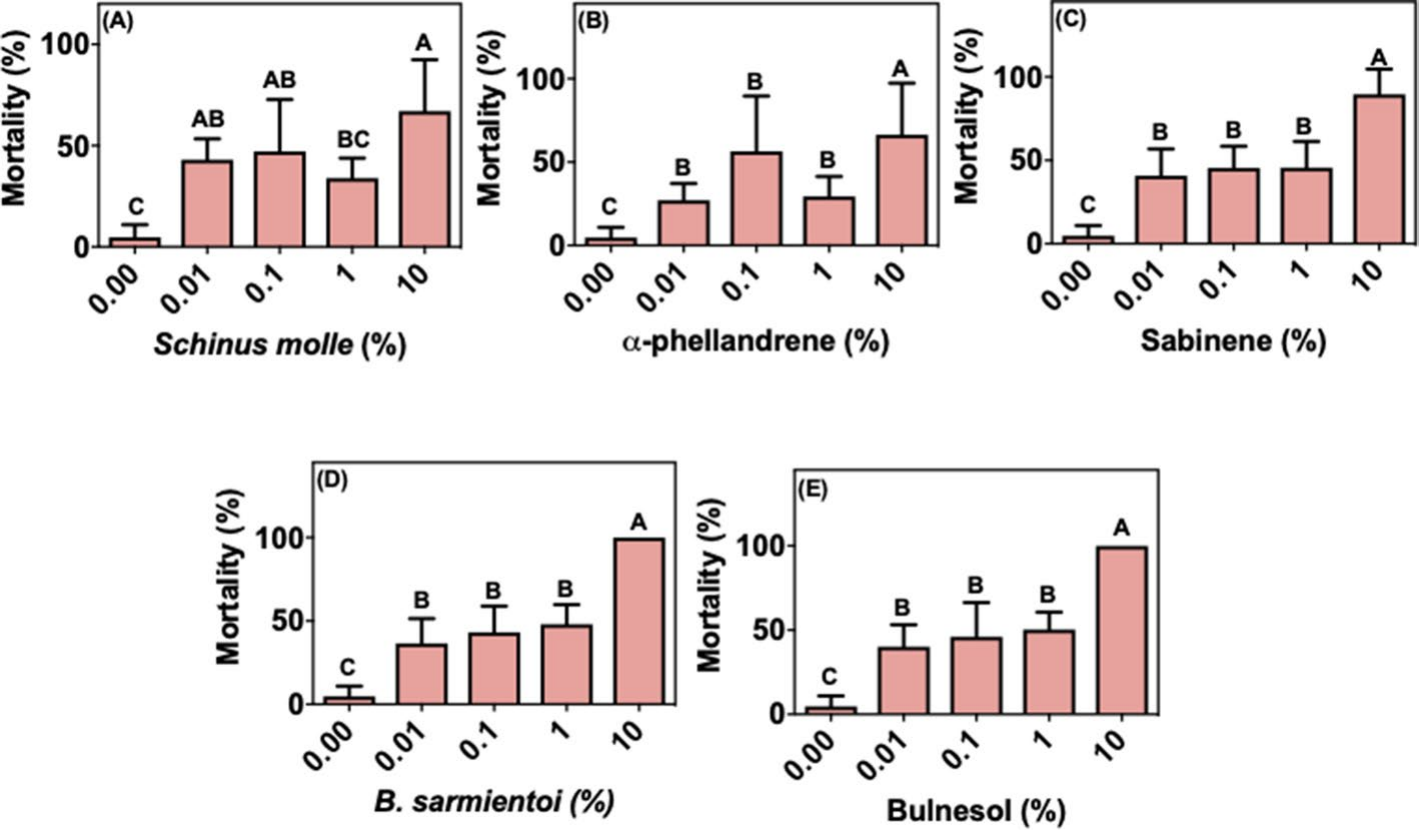
Mean (+ SD; n = 3) mortality of larvae from *Rhipicephalus pulchellus* African field population after exposure to essential oils and their main components. **a** *Schinus molle* essential oil, **b** α-phellandrene, **c** sabinene, **d** *Bulnesia sarmientoi* essential oil, **e** bulnesol, and **f** guaiol. Means capped with the same letter are not significantly different (Tukey test: p > 0.05)

For *R. pulchellus*, *S. molle* essential oil, α-phellandrene and sabinene did not reach more than 50% of larval mortality at concentrations between 0.01 and 1%. At 10%, sabinene caused 90% of larval mortality (Fig. 5c), whereas α-phellandrene and the essential oil reached a mortality of < 70% at the same concentration (Fig. 5a, b). Treatment with *B. sarmientoi* essential oil and bulnesol caused 100% larval mortality at the highest concentration tested (Fig. 5d, e).

**Fig. 6:**
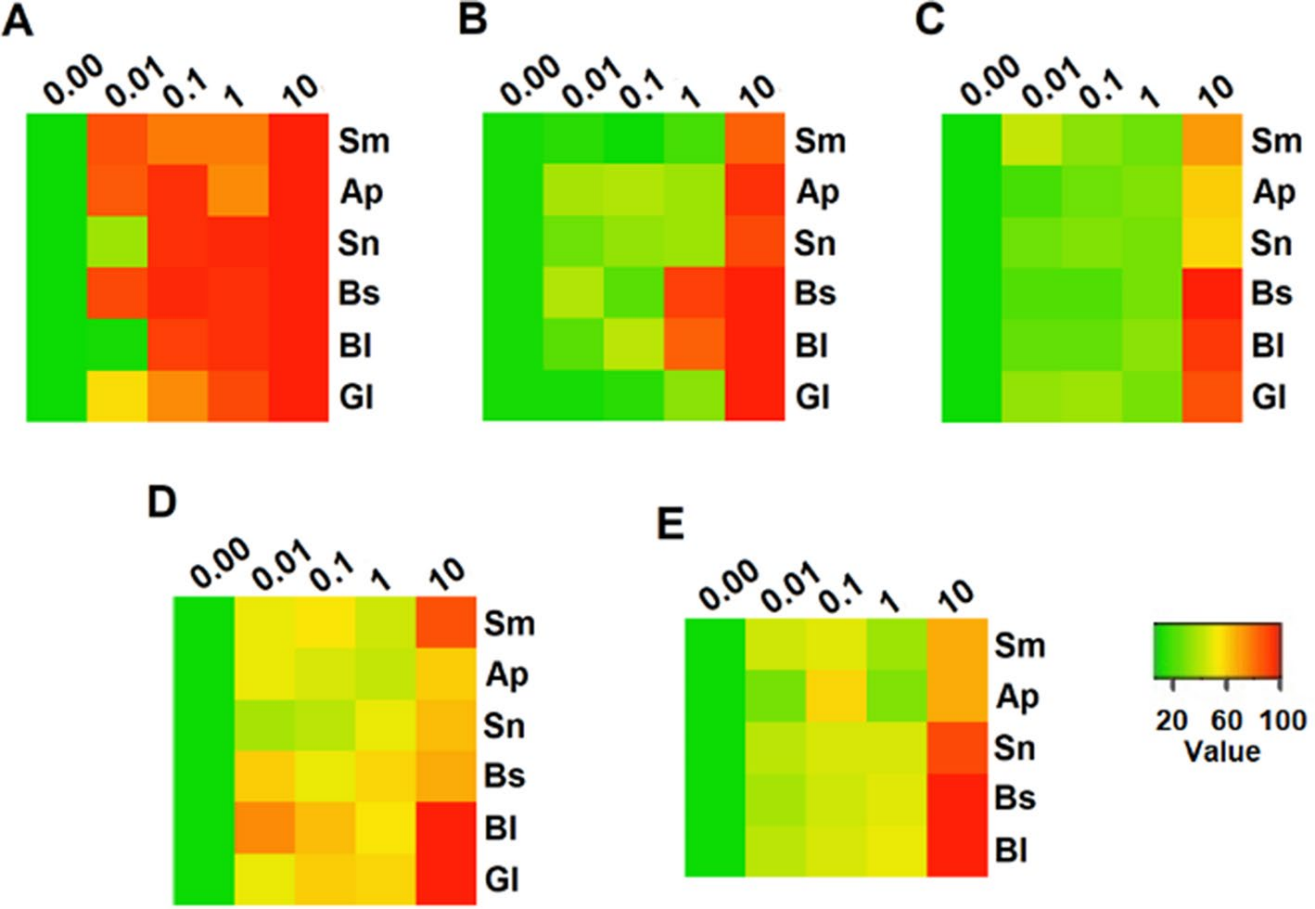
Heat map of essential oil effects on *Rhipicephalus* spp. Data are presented as percentage mortality (0–100%) after treatment with essential oils or isolated compounds at concentrations from 0.01 to 10% in larval immersion tests of **a** laboratory maintained *R. microplus* strain; **b** *R. microplus* field population; **c** *R. evertsi* wild population; **d** *R. appendiculatus* wild population; and **e** *R. pulchellus* wild population*.* The map shows the drastic difference in xenobiotic resistance between samples with or without history of acaricide exposure, and the resistance in African ticks. Sm, *Schinus molle* oil; Ap, α-phellandrene; Sn, sabinene; Bs, *Bulnesia sarminetoi* oil; Bl, bulnesol; Gl, guaiol

Mortality after the various treatments is summarized as a heat map (Fig. 6). It shows that the essential oils and their components are similarly active against African ticks (*R. evertsi*, *R. appendiculatus* and *R. pulchellus*) and the *R. microplus* field population (Fig. 6b–e). *Rhipicephalus microplus* from the Porto Alegre strain presented a greater sensitivity to essential oils and their components when compared to African ticks, despite being equally free of previous acaricide exposure (Fig. 6a).

## Discussion

Cases of resistant tick populations started to be reported in 1937 for arsenical acaricides, and nowadays there is resistance to all known classes of acaricide (Angus 1996; George et al. 2008; Rodriguez-Vivas et al. 2018). In the present study, it was observed that *R. microplus* ticks previously exposed to acaricides (Fig. 2) were less susceptible to treatment with essential oils and their selected compounds, whereas ticks that have never been exposed to acaricides suffered high larval mortality upon these treatments (Fig. 1). However, African ticks without contact with acaricides displayed a mortality profile closer to the acaricide-resistant field *R. microplus* strain, as opposed to the susceptible Porto Alegre strain.

In the present study we show that the *R. microplus* field population has lower susceptibility to essential oils and its components when compared to a strain that did not suffer the same selective pressure (Porto Alegre strain) (Figs. 1, 2; 6a, b). The immersion test with *S. molle* and *B. sarmientoi* essential oil revealed an unexpected tolerance in *Rhipicephalus* ticks from East Africa (without acaricide exposure) almost higher than in the Brazilian strain with a history of exposure to acaricide (Figs. 2a, d; 3a, d; 4a, d; 5a, d; 6).

The toxicity of the essential oils used in this study against various arthropod species was also documented by other researchers. The *S. molle* essential oil presented repellent and insecticidal activity against *Trogoderma granarium* and *Tribolium castaneum* with mortality rates of 90 and 76.7%, respectively, at a concentration of 10% (Abdel-sattar et al. 2010). The biological activity observed in the essential oil of *S. molle* can be attributed to the concentrations of α-phellandrene and sabinene (Matias et al. 2016; Zhang et al. 2017). Hexane extracts from leaves and fruits of *S. molle* also had a repellent effect on *Triatoma infestans* and *Cydia pomonella* larvae (Ferrero et al. 2006). The repellent activity found for Oriental cockroach (*Blatta orientalis*) was attributed to the major components (germacrene D and β-caryophyllene) (Deveci et al. 2010). *Schinus molle* essential oil presented a high level of toxicity against *Artemia salina*, with LC_50_ values of 47 and 67 mg/ml for leaf and fruit, respectively (Martins et al. 2014). Other parasites of economic importance are also affected by these essential oils. For instance, the flea *Ctenocephalides felis*, the most important ectoparasite of dogs and cats, as well as the cattle tick *R. microplus* showed high mortality after treatments with fractions of this essential oil, in line with the present results (De Batista et al. 2016; Torres et al. 2012). *Rhipicephalus microplus* without previous acaricide exposure were tested using *S. molle* essential oil in a larval immersion test, and the mortality rate was similar to the one observed for the field strain in the present study (Torres et al. 2012).

In African species, substances isolated from *B. sarmientoi* essential oil—guaiol and bulnesol—were not as active as was the original essential oil, suggesting a synergistic effect between these and other substances from the plant. In the present study, this essential oil induced 100% larval mortality for all tested tick species at 10% concentration, demonstrating that, besides being able to inhibit tick feeding, *B. sarmientoi* also presents acaricide action (D panels in Figs. 1, 2, 3, 4, 5, and 6). Further studies on the effect of *B. sarmientoi* on other arthropods and mammals are needed to assess their specificity and the possibility for the development of new products for tick control.
